# Diagnostic performance of black tent sign on 3D T2 SPACE in the diagnosis of idiopathic normal-pressure hydrocephalus

**DOI:** 10.1007/s11604-025-01757-x

**Published:** 2025-03-08

**Authors:** Ahmet Yalcin, Tugrul Akkus, Alperen Tezcan, Birkan Usta, Revza Yalcin, Fatma Simsek, Mete Zeynal

**Affiliations:** 1 Faculty of Medicine, Department of Radiology, Ataturk University, H.Avni Ulas M. Haznedar C. Sagsoz Apt. A blok. No: 12, 25240 Erzurum, Turkey; 2 Section of Radiology, Battalgazi State Hospital, Malatya, Turkey; 3 Faculty of Medicine, Department of Neurology, Ataturk University, Erzurum, Turkey; 4 Faculty of Medicine, Department of Neurosurgery, Ataturk University, Erzurum, Turkey

**Keywords:** Idiopathic normal-pressure hydrocephalus, T2 SPACE, Diagnosis, Black tent

## Abstract

**Purpose:**

Idiopathic normal-pressure hydrocephalus (iNPH) presents with Hakim’s triad and diagnosis is solely based on clinical findings. The role of imaging is confined to the detection of ventriculomegaly and the exclusion of other possible entities. Hyperdynamic CSF flow has been demonstrated in various flow-related imaging studies. In this study, we aimed to investigate the diagnostic performance of the “black tent” sign in the CSF flow-sensitive T2 SPACE sequence.

**Materials and methods:**

This retrospective study includes 22 patients diagnosed with iNPH who underwent CSF shunting and benefited from the procedure and showed clinical recovery. The control group consisted of 38 patients with excluded diagnoses of iNPH by clinical examination and follow-up. T2 SPACE images from both groups were assessed according to the presence of the “black tent” which was defined as a signal void detected on the T2 SPACE image traced along the borders of the fourth ventricle and filling the triangular area of the median dorsal recess. The diagnostic performance of the sign was calculated, and the results were compared with those of Evan’s Index, callosal angle, and disproportionately enlarged subarachnoid spaces.

**Results:**

The diagnostic performance of the black tent sign in diagnosing iNPH was determined with a sensitivity of 90.91%, specificity of 78.95%, PPV of 71.43%, NPV of 93.75%, and overall accuracy of 83.33%. The sign showed better diagnostic performance in participants over 60 years in which sensitivity, specificity, PPV, NPV, and accuracy increased to 86.67%, 93.75%, 86.67%, 99.75%, and 91.49% respectively. Diagnostic performance of the sign was superior to DESH (*p* = 0.007).

**Conclusion:**

The black tent sign observed in T2 SPACE images in CSF flow MRI studies correlates with the diagnosis of iNPH with high sensitivity and specificity.

## Introduction

Normal pressure hydrocephalus (NPH) is a clinical disease presenting with Hakim’s triad which is composed of gait disturbance, cognitive decline, and urinary incontinence [1, 2]. NPH is classified as idiopathic and secondary. Imaging findings play an important role in making a distinction between idiopathic and secondary NPH. In recent practice, NPH is diagnosed when two out of three symptoms of Hakim’s triad are present; however, these symptoms are generally absent in the early course of the disease. The imaging findings are essential in diagnosis as the symptoms are non-specific. The main imaging finding of idiopathic NPH (iNPH) is ventriculomegaly; however, ventriculomegaly can be seen because of normal aging, infarct, previous neurosurgery operation, or as a sequel of trauma [3].

Recent guidelines proposed that disproportionately enlarged subarachnoid spaces (DESH) and Evan’s Index (EI) are the morphological criteria for radiological diagnosis [2]. Ventriculomegaly with EI >0.3 in association with high convexity subarachnoid tightness and sylvian fissure enlargement were defined as DESH findings [4]. Some shortcomings related to diagnosis based on these findings were also pointed out in these guidelines. iNPH is also classified into three categories namely possible, probable, and definite iNPH [2]. Possible iNPH patients are defined as having more than one symptom of the clinical triad which cannot be explained by any other condition and there is no identifiable cause for ventriculomegaly [2]. The diagnostic criteria for probable iNPH are as follows: CSF pressure of less than 200mm H_2_O and normal CSF content in patients meeting possible iNPH criteria and improvement of symptoms after CSF tap test or DESH in MRI [2]. Definite iNPH refers to patients who respond to shunt surgery [2].

Several studies have shown increased cerebrospinal fluid flow parameters in the pathophysiology of iNPH [5, 6]. Nowadays, conventional magnetic resonance imaging (MRI) or computed tomography (CT) is used to demonstrate ventricular dilatation and to distinguish between idiopathic and secondary NPH [2, 7]. As the symptoms are non-specific, CSF flow studies have come into use to demonstrate CSF flow dynamics in iNPH patients. Many studies show increased CSF flow in iNPH patients; however, there is no single parameter or cut-off value to make the diagnosis. Despite the numerous studies on CSF flow parameters, the definite diagnosis of iNPH is made with a CSF tap test or with remission of symptoms after CSF shunting [8]. Early diagnosis of iNPH is very important, because the chance of symptom remission is decreased when irreversible neurodegeneration develops [3].

In this study, we investigated the diagnostic efficacy of the signal void filling the entire fourth ventricle in CSF flow-sensitive T2 SPACE images as hyperdynamic CSF flow constitutes the pathophysiology of the disease. We postulate that hyperdynamic and chaotic CSF flow as implied by these imaging phenomena is due to the deterioration of normal pulsatile CSF flow. We aim to seek its presence in patients with iNPH and controls then calculate its diagnostic performance and compare it to those of DESH, EI, and callosal angle.

## Materials and methods

### Patients

This study was approved by the institutional ethics committee. Informed consent was waived due to the retrospective nature of the study. A total of 438 patients who were referred to our radiology department for MRI CSF flow studies with provisional diagnosis of iNPH between February 2018 and February 2023 were included in this study. From this patient population, 32 patients were included as the initial iNPH group who had the classical triad of symptoms. From this group of patients, 4 patients were excluded because of rejecting the lumbar puncture procedure; 5 patients were excluded because of not showing improvement after the lumbar puncture, and one patient was excluded because of rejecting the permanent shunt placement. Thus, 22 patients with an established diagnosis of iNPH with response to the CSF shunting were chosen as the iNPH group.

**Fig. 1:**
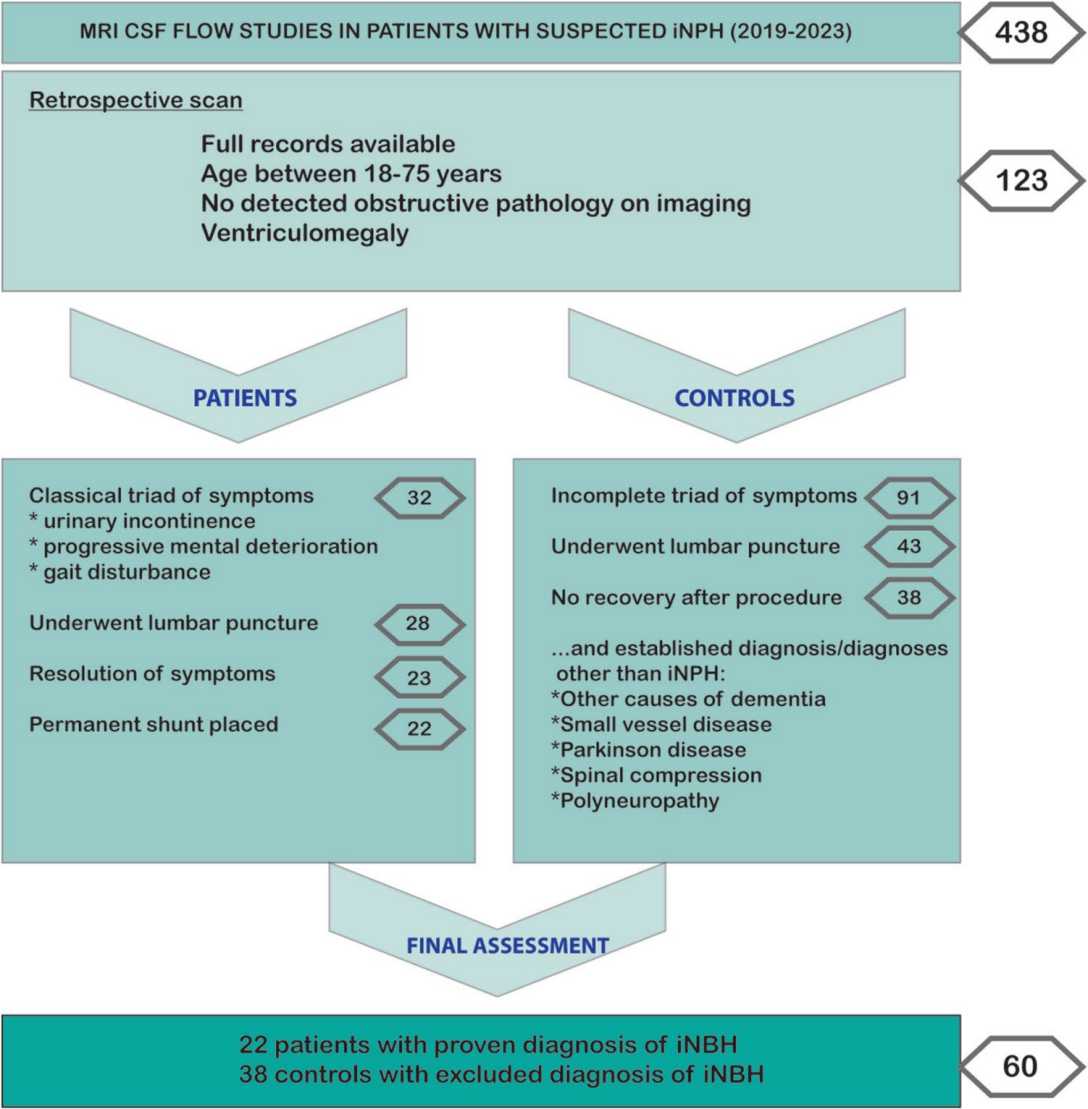
Flowchart of the study with inclusion and exclusion criteria

A total of 91 patients who were presented with 2 symptoms of Hakim’s triad at most were designated as the control group. From this population, 48 patients were excluded because of rejecting the lumbar puncture procedure, and 5 patients were excluded because of showing minimal improvement in symptoms. Thus, 38 patients with non-communicating hydrocephalus and clinical exclusion of NPH were chosen as the control group. The final diagnoses of the control group were listed. A study flowchart with inclusion criteria is presented in Fig. 1.

### CSF flow study and image analysis

All MRI assessments were performed using a 3T scanner (Aera, Siemens Healthcare, Erlangen, Germany) before the CSF shunting. CSF flow studies consisted of sagittal volumetric T2 SPACE along with sagittal T2 CISS sequences. Imaging parameters for T2 SPACE were as follows: TR 3200 ms, TE 411 ms, voxel size 1.0 x 1.0 x 1.0 mm, Field of view (FOV) 250mm, slice thickness 1.00 mm. Variable flip angle mode was selected as the “T2 variable” under the sequence settings.

**Fig. 2:**
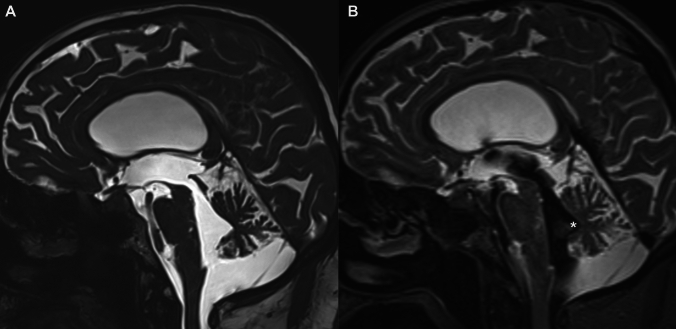
**AB** Sagittal T2-weighted CISS (**A**) and SPACE images (**B**) in a patient with iNPH. Note the presence of ventriculomegaly in lateral ventricles and hyperdynamic CSF flow in the fourth ventricle associated with marked signal void. Hyperdynamic CSF flow is also visible in the median dorsal recess (white asterisk)

All images were transferred to the offline workstation. T2 SPACE sequences in CSF flow MRI studies were evaluated by two independent radiologists (AY and AT) retrospectively without access to patients’ clinical information. Multiplanar reconstruction was performed to obtain a coronal image through the posterior commissure, perpendicular to the anterior–posterior commissure plane. The callosal angle was measured as the angle between the lateral ventricles on the coronal images [9]. Axial reconstructed T2 SPACE MRI was used to calculate EI. The maximal width of the frontal horns of the lateral ventricles was divided by the maximum inner diameter of the skull on the same slice [10]. DESH was diagnosed on coronal T2 SPACE images as disproportionate enlargement of the inferior subarachnoid spaces mainly the sylvian fissures with tight high convexity subarachnoid spaces [4]. The black tent sign was defined as a smooth signal void detected on the T2 SPACE image which is traced along the borders of the fourth ventricle and completely fills the triangular area of the median dorsal recess (Figs. 2AB and 3AB). For each patient, the presence or absence of the black tent sign was noted. Imaging findings were then compared to the post-surgical clinical findings which are considered the gold standard.

**Fig. 3:**
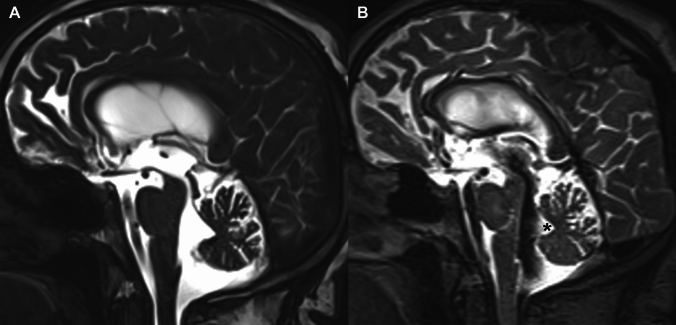
**AB** Sagittal T2-weighted CISS (**A**) and SPACE images (**B**) in a patient without iNPH. Lateral ventricles were also dilated in this patient. A thin line of signal void is seen within the fourth ventricle associated with normal CSF flow. Note the absence of flow at the level of the median dorsal recess (black asterisk)

### Statistical analysis

The distribution of normality was assessed with the Shapiro–Wilk test. Summary statistics were reported as mean± SD for normal and median (IQR) for the non-normal distributions. Continuous variables with normal distribution were compared with an independent t test, whereas those with non-normal distributions were compared using Mann–Whitney U test. Nominal categorical variables were assessed with the Chi-square test. Inter-rater reliability was determined using Cohen’s kappa coefficient with linear weights. Comparison of diagnostic performances belonging to two different methods was performed using the McNemar test. ROC analysis was performed to detect the AUC and Youden Index. A two-tailed p value < 0.05 was accepted as statistical significance. Inferential statistics were analyzed via the R statistical software package (R studio ver. 4.3.2, Vienna, Austria).

## Results

The mean age of the study population was 66.5 ± 15.5 years (range 33–89 years) and 32 out of 60 participants were male (53.3%). Twenty-two participants diagnosed with iNPH showed relief of symptoms after the CSF drainage. The control group consisted of 38 patients with non-communicated hydrocephalus with no established diagnosis of iNPH.

**Fig. 4:**
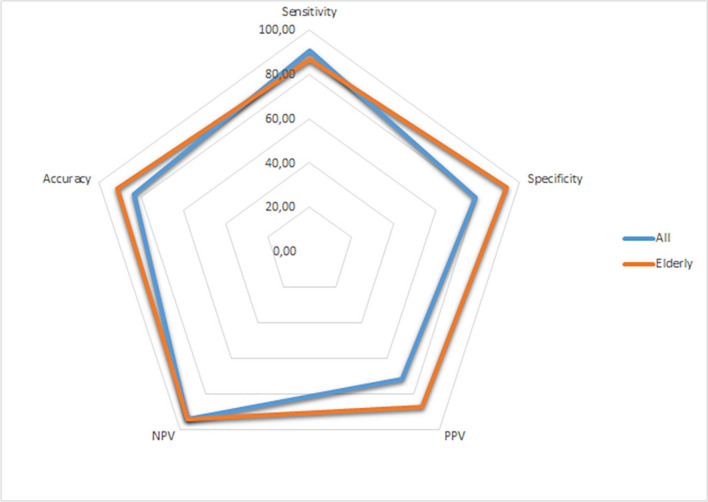
The radar graphic shows the diagnostic performance of black tent sign in all patients (blue) and elderly patients only (orange)

The black tent sign was present in 20 of the 22 patients (90.9%) with iNPH, whereas it was present in 8 of 38 patients (21.1%) with no iNPH. The demographic and study data are summarized in Table 1. The diagnostic performance of the black tent sign in the diagnosis of iNPH was determined with a sensitivity of 90.91%, specificity of 78.95%, PPV of 71.43%, NPV of 93.75%, and accuracy of 83.33%. The sign showed better diagnostic performance in participants over 60 years in which sensitivity, specificity, PPV, NPV, and accuracy changed to 86.67%, 93.75%, 86.67%, 93.75%, and 91.49% respectively (Fig. 4). Mean age of the false positives in control group and false negatives in iNPH group were 44.8 ± 22.8 and 73.0 ± 9.9 years, respectively.

**Table 1:** Demographic and study data in patients and controls with corresponding p values

	iNPH group (*n* = 22)	Controls (*n* = 38)	*p* value
Age, years ±SD	64.7 ± 12.7	67.6 ± 17.0	0.490
Gender, male (%)	11 (50)	21 (55.3)	0.696
Black tent sign (%)	20 (90.9)	8 (21.1)	**<0.001**
DESH (%)	7 (31.8)	6 (15.8)	0.259
Mean callosal angle ±SD	92.88 ± 23.11	108.21 ± 19.36	0.012
Median Evans index (IQR)	0.315 (0.047)	0.325 (0.040)	0.578

Bold text denotes the statistical significance less than 0.05

*iNPH* idiopathic normal-pressure hydrocephalus; *SD* standard deviation; *DESH* disproportionately enlarged subarachnoid spaces; *IQR* interquartile range

Strong agreement was observed between two observers according to kappa statistics (ĸ = 0.82, 95CI = 0,648–0,988).

Diagnostic performance of DESH was determined with a sensitivity of 31.82%, specificity of 84.21%, PPV of 53.85%, NPV of 68.09%, and accuracy of 65%. Black Tent sign had better diagnostic performance compared to DESH (*p* = 0.007).

Callosal angle (cut-off value: 90.7) and Evans index (cut-off value: 0.29) had a sensitivity of 81.57% and 95.45%, specificity of 59.10%, and 18.43% and AUC of 0.693 and 0.543 respectively.

## Discussion

In this study, we have described a unique imaging finding that accurately estimates the diagnosis of iNPH in a suspected clinical setting. The sensitivity of the black tent sign in the diagnosis of iNPH was 90.91% and the negative predictive value (NPV) was found to be 93.75% which demonstrates its value in the exclusion of iNPH from other possible overlapping diseases. The success rate of black tent sign became even higher in older patients.

Normal pressure hydrocephalus is a rare cause of dementia that can be cured with surgery. However, only patients with hydrocephalus benefit from CSF shunts, and it is hard to make the final diagnosis [3]. Clinical and imaging findings overlap with various entities, and therefore, there are numerous studies on advanced diagnostic modalities [3]. The lumbar infusion test and tap test are beneficial in diagnosis with varying sensitivity and specificity. Also, there is a risk of infection associated with this procedure mentioned in several studies [3]. Conventional MRI is used to exclude other pathologies that overlap symptoms with iNPH. The imaging features that are not diagnostic but support the diagnosis were as follows: disproportionately enlarged subarachnoid space, narrowing of the sulci and subarachnoid spaces over high convexity, ventricular enlargement disproportionate to cerebral atrophy, periventricular hyperintensities, and temporal horn dilatation without evident hippocampal atrophy [11]. Some imaging-based calculations such as EI are used as a screening tool to determine hydrocephalus. A recent meta-analysis and systematic review demonstrated that EI should be used for screening for ventricular enlargement in iNPH patients [10]. On the other hand, it is shown that ventricular enlargement is predominantly on the z-axis rather than the x-axis in iNPH patients [12]. A study performed by Yamada et al. showed that expansion of the lateral ventricles in the z-axis is a common parameter to distinguish NPH from Alzheimer’s disease [13]. In our study, EI had poor diagnostic performance in the detection of iNPH in patients with ventriculomegaly.

In patients with definite iNPH, DESH findings were reported in 64%, and non-DESH findings were seen in 36% [2]. In our study, 20 out of 22 (90.9%) definite iNPH patients had black tent sign, whereas 7 out of 22 (31.8%) patients had DESH findings. The sensitivity of DESH was lower in our study compared to that reported in the literature. DESH was reported to have a 25% NPV in the diagnosis of iNPH in a study carried out by Craven et al. [14] NPV of the DESH was 68.1% in our study. It was NPV of the DESH was found to be higher in our study compared to literature. Nevertheless, the black tent sign showed better diagnostic performance compared to DESH with higher sensitivity, PPV, NPV, and accuracy.

A meta-analysis of examining the diagnostic performance of callosal angle included 874 patients; the pooled sensitivity and specificity of the callosal angle in the diagnosis of iNPH were 91% and 93%, respectively [15]. The diagnostic performance of the callosal angle was lower compared to the black tent sign in our study (81.57% vs. 90.12% for sensitivity and 59.10% vs. 78.95% for specificity respectively).

Recent guidelines only use radiological signs of ventricular dilatation apart from clinical findings to interpret impaired CSF clearance which is the pathophysiology of iNPH.

There are many studies demonstrating increased CSF flow parameters at the level of the cerebral aqueduct. However diagnostic efficacy of these findings varies between studies [3]. There is no cut-off value for the diagnosis of iNPH as there is no large-scale study. In studies that obtain a cut-off value, there is a comparison between iNPH patients and healthy individuals, so there is no parameter to distinguish iNPH patients with overlapping diseases [3]. The theory on the pathophysiology of iNPH suggests that the mechanical stress on the periventricular white matter is caused by ventricular dilatation which causes ischemia and the hypoxia of the axons [16]. The ependymal layer loses plasticity and loses its ability to transmit pulsations; thus, CSF flow through compartments is impaired [16]. Several studies on CSF dynamics with phase-contrast MRI were done to obtain diagnostic criteria. Unfortunately, studies showed that phase-contrast MRI is hard to interpret, and the results vary according to the MRI scanner used. [17]. Peak mean velocity and aqua ductal stroke volume are mostly studied parameters, but the results are controversial. A study carried out by Tawfik et al. shows a diagnostic accuracy of 92.5%–93.3% for peak mean velocity and even 100% for aqua ductal stroke volume [18]. However, the aqua ductal stroke volume was shown to be a poor predictor of shunt response by Blitz et al. [19]. There is no consensus on objective radiologic diagnostic criteria for iNPH. We have postulated that hyperdynamic and chaotic CSF flow as implied by these imaging phenomena is due to deterioration of the normal pulsatile CSF flow. We think that this altered flow dynamics affects the CNS negatively in two ways. First, this type of flow change might reduce the functions of the glymphatic system as the clearance of solute excess molecules which are normally enhanced by pulsatile flow [20]. This explains the mechanism of the symptoms associated with cognition as the link has already been suggested [21]. The second mechanism that we associate with this imaging finding is the presence of such CSF vortex within the fourth ventricle might insult the ependymal layer as we mentioned earlier. At the level of the medial dorsal recess of the cerebellum lies the fastigial nucleus. Thus, minor repetitive fluctuations occurring at this location without increased pressure might affect the functions of the fastigial nucleus which has an important role in motor coordination and micturition [22].

Some clinical implications should be considered in this study. The presence of the “black tent” sign was associated with the diagnosis of iNPH in our study. In addition to its diagnostic capability, the sign was also found to be useful for the prediction of the benefit of CSF drainage due to our patient selection criteria in which they were selected not only for the presence of classical symptoms of iNPH but also for the presence of treatment response.

Some limitations should also be considered in our study. First, the accuracy of this imaging feature should be tested in larger study groups. Second, the diagnostic capability of this sign seems valid in older patients as the CSF circulation seems already hyperdynamic in younger patients. This is why, we had higher false-positive results in the whole patient population compared to elderly patients. Third, the performance of the sign was tested in patients with a suspected diagnosis of iNPH, and thus, diagnostic performance without pretest probability should also be assessed in further studies.

In conclusion, the “black tent” sign seen in T2 SPACE images in CSF flow MRI studies correlates with the diagnosis of iNPH with high sensitivity and specificity. Furthermore, the sign is associated with treatment response owing to our patient selection criteria.
